# Supplementary material for: Are carbon emissions trading and green financial instruments synergistic? -Comprehensive quantitative research based on content analysis
Source: PLoS One. 2024 Mar 7;19(3):e0298601. doi: 10.1371/journal.pone.0298601 (PMC10919601; doi:10.1371/journal.pone.0298601)
Supplement: S1 Data — (ZIP) [file pone.0298601.s001.zip › Data/地级市Z_F工作报告.zip!/╕ú╜¿/╞╬╠∩╩╨/└┤╫╘╣1⁄2╓┌║┼í╛┬φ┐╦╩2╛▌═°í┐_2016.docx]

各位代表：

　　现在，我代表莆田市人民政府，向大会报告政府工作，请予审议，并请市政协委员提出意见。

　　一、2015年和“十二五”时期工作回顾

　　2015年，在省委、省政府和市委坚强领导下，我市各级政府深入学习贯彻党的十八大、十八届三中、四中、五中全会和习近平总书记系列重要讲话精神，全力稳增长、调结构、促改革、惠民生、防风险，经济社会平稳健康发展。初步预计，全市生产总值1655.2亿元、增长10.5%；财政总收入185.2亿元、增长5.8%；地方财政收入115.7亿元、增长4.8%；全社会固定资产投资1765.1亿元、增长21.5%；外贸出口总额31.7亿美元、下降3.6%；实际利用外资3.8亿美元、增长10.7%；社会消费品零售总额558.9亿元、增长12.2%；居民消费价格总水平上涨1.5%；居民人均可支配收入2.2万元、增长8.6%；城镇登记失业率2.2%；人口自然增长率7.5‰；年度节能减排任务全面完成。受经济下行、“营改增”、企业欠税、价格因素等影响，全市生产总值、财政总收入、外贸出口总额、农村居民人均可支配收入等指标与预期有差距。

　　一年来的主要工作和成效是：

　　（一）经济平稳健康增长。认真落实稳增长政策，开展“进企业、摸实情、解困难”活动，召开产业转型升级系列沙龙，因企施策、一企一策，出台工业稳定增长创新转型、扶持小微企业、促进政府融资性担保机构发展、加快鞋业、木材、工艺美术转型、重点出口行业转型升级等政策，稳住经济基本面,三次产业比例由上年的7.3:57.7:35.0调整为6.9:57.4:35.7，经济结构进一步优化。金融服务实体经济有效提升，新增3家金融机构，创新“无间贷”、“连连贷”等金融产品，金融机构本外币存款余额1608.2亿元、增长10.7%，金融机构本外币贷款余额1466.2亿元、增长9.8%；支持企业直接融资，三棵树IPO通过证监会批准，国德医疗在新三板挂牌，海丝商品交易中心获批;设立转贷资金，为297家企业转贷应急资金147亿元，处置不良贷款45.3亿元，担保圈贷款风险防控取得实效。新设鞋业成长互助资金，互助资金增加至9支，为3019家企业发放产业互助成长资金贷款10.71亿元。农林牧渔业增加值124.3亿元、增长2.2%，推广农业标准化生产和“利农模式”，实施设施农业项目40个，新增设施果蔬6千亩、楼房养猪4万头、大棚养禽100万羽，主要粮食作物生产机械化综合水平达42%。规上工业增加值799.1亿元、增长10.8%，新增规上工业企业175家，高技术企业增加值增长10.8%，工业用电量53.4亿千瓦时、增长31.3%，PA6、华峰纺织科技产业园等竣工投产，高世代面板、CPL、雪津迁建、华源纺织等开工建设，和润粮油、新能源汽车、威高医疗器械等前期工作有序推进。第三产业增加值590.7亿元、增长12.1％，实现营利性服务业增加值127.4亿元、增长26.5%，新增规上服务业企业113家；举办“中国质造”系列活动，创建普天药械网、找银网、中房购等电商平台，新增限上电商企业62家，销售额33.6亿元、增长172.0％；落实房地产稳增长政策，货币安置取得突破，房地产销售295.7万平方米、增长14.3%；湄洲岛游客量突破400万人次，全市旅游总收入168.9亿元、增长24%。

　　（二）项目推进成效明显。实行重大项目“两周一调度”、协调问题“一周一报告”、效能督查“一季一通报”、项目征迁和要素保障“一把手”负总责等机制，成立重大项目服务组，推广涵江征迁经验，项目带动有力有效。设立电子信息产业股权投资基金25亿元，争取地方债券148.6亿元、专项基金11.1亿元。500个重点项目完成投资1193.7亿元，超额完成年度计划。国投配煤基地铁路支线、平海湾海上风电等项目实现开工，湄渝高速莆田段、金钟水利枢纽引水配套工程等项目实现竣工，联十一、联二、滨海大道等项目加快推进，宁海闸等项目前期工作取得突破。实施省市重点技改项目115个，投产35个。仙游至长乐城际轨道、南日岛海上风电40万千瓦项目获批，仙游抽水蓄能电站一期获“国家优质工程金质奖”。通过盐田置换方式，废转盐田1860亩，为项目建设腾出空间。加强与央企、省企战略合作，积极引进港澳台侨外大企业，举办3场民企产业链招商会；全年共签约央企、民企项目43个、总投资1188亿元，外企项目20个、总投资18.7亿美元。

　　（三）宜居城乡加快建设。城乡基础设施建设不断推进，完成投资341.3亿元。市政方面，新建改建道路124条，新建扩建污水厂3个，闽中污水处理厂三期投入使用，新增污水日处理能力9.9万吨，配套污水管网210公里，全市污水处理率达85%。扩建生活垃圾焚烧发电厂。重点工业园区完成基础设施投资85.9亿元，增长18.6%；入驻企业1576户，完成工业固定资产投资380亿元，增长5.9%。交通方面，新建续建泊位14个，新增港口吞吐能力1698万吨，新扩建6个陆岛交通码头。新增公交车、出租车461辆，公交占机动化出行比例达16.5%。水利方面，新建续建重大水利项目36个，木兰溪防洪工程、乌溪水库、妈祖城水厂、金莆供水工程等加快建设，加快推进城乡供水一体化。城市管理方面，深入实施宜居环境建设行动计划，整治城市内河80公里，拆除“两违”建筑226.7万平方米，改造建设公园景区16个，建成高标准绿道39公里，整顿交通秩序，市民素质持续提高，获全国文明城市提名。

　　（四）生态环境持续改善。淘汰小锅炉82台，全市单位生产总值能耗下降5.9%。绿化造林7.75万亩，森林覆盖率达59.8%，桉树择伐试点成效明显，提前完成“十二五”“双控”任务。4个县区获评省级生态县，22个乡镇通过国家生态乡镇验收。列入全国水生态文明城市建设试点，拆除关闭畜禽养殖场57.5万平方米，综合治理东圳水库，饮用水源地水质明显改善。开展农村生活垃圾治理专项行动，有力改善农村环境。全面实施机动车环保标志管理，淘汰黄标车3345辆，环保标志核发率90%以上，空气质量达标天数比例97%以上，居全省前列。

　　（五）改革开放不断深入。新型城镇化、民营经济、电子商务、新能源、智慧城市、信息惠民、公立医院、社会资本办医八项国家改革试点有序开展。市行政服务中心列入国家第二批社会管理和公共服务综合标准化试点。市政道路工程等11类采取并联审批，食品药品实行“九证合一”，工商质监税务施行“三证合一”、“一照一码”登记制度。在省内率先启用“社保通”自助服务平台。7个县区（管委会）行政服务中心、54个乡镇便民服务中心设立实体办事大厅，建成村居便民服务点820个，形成覆盖市县乡村四级服务体系。城乡一体化综合配套改革取得突破，“幸福家园”十镇百村投资9.7亿元，发展农民合作社1416家，流转土地15.5万亩，“两证合一”确权发证全面展开。完成15个村改居，全市常住人口城镇化率达57%。成立莆阳资产管理公司，党政机关与所办企业全部脱钩。推进公立医院改革，开展分级诊疗试点，实行药品、耗材零差率销售，减轻群众就医负担。采取民营投资、托管合作模式，推进市中医医院、涵江华侨医院、华亭卫生院改革，南日岛、湄洲岛卫生院由市两大医疗集团直管，职工收入增加，服务水平提升，群众得到实惠。智慧城市建设加快，发放城市一卡通26万张，与全国78个城市互联互通。举办“第六届世界兴安同乡恳亲大会”、“第七届世界莆商大会”、“华侨华人项目成果专场对接会”、东南亚经贸活动周等活动。莆台交流合作取得新成效，新批台资项目12项、总投资2.3亿美元，莆台联创歌曲《两岸一家亲》在第七届“海峡论坛”上唱响。与澳大利亚帕拉玛塔市缔结友好城市。

　　（六）社会事业积极发展。15项为民办实事项目基本完成年度任务。新增公办幼儿园12所。新建、扩建义务教育学校16所，新增中小学学位1.1万个，改革义务教育招生，4500多名农民工子女就读城区公办义务教育学校。医疗卫生服务体系不断健全，新增床位800张。筹办健康产业洽谈会，健康产业园积极推进。采取政府购买商业保险方式经办新农合，（上接Ａ１版）城乡居民基本医疗保险人均筹资水平提高到470元，参保率达99%。率先在全省实现城乡低保、抚恤补助、低保户小额保险一体化，发放各类社会救助资金4.35亿元，荔城区“救急难”工作被列为全国试点。城乡居民基础养老金从每人每月70元提高到85元，新建农村居家养老幸福院32个。农民工职业技能培训6000多人次，新增就业人数2.22万人。保障性安居工程开工1.44万套、竣工6400套，棚户区项目改造31个、棚改安置房完成1.37万套，安置房办证1.5万套。新建文化激情广场30个、数字农家书屋100个。建成仙游大剧院。参与海丝世界文化遗产申报。省第六届艺术节获奖57个，莆仙戏《魂断鳌头》获省戏剧会演优秀剧目一等奖。《春草闯堂》选段参演中宣部、文化部新年戏曲晚会。成功举办妈祖文化旅游节、央视“心连心”艺术团演出、中国红木艺雕精品博览会、第五届菊花展、第五届南少林武术文化节、南日岛全国海钓锦标赛和全国首届青运会莆田赛事等文体活动，获得各类体育奖项46项。完成村居换届，两委女性比例全省第一。严厉打击违法犯罪，加强社会治安防控，“三化一龙头”工作走在全省前列，社会安定稳定。“六五”普法顺利完成，办理法律援助案件8000多件，调解各类矛盾纠纷4万多件。阳光信访、法治信访、责任信访有序推进，非访数量下降，信访“三率”提高，积案有效化解，信访工作水平不断提升。全力抗击“苏迪罗”、“杜鹃”等强台风正面袭击，快速开展灾后重建，实现“不死人、少损失”目标。安全生产事故起数、死亡人数全面下降。工会、共青团、妇联、档案、地方志、残疾人、红十字等工作取得新进步。

　　（七）政府自身建设继续加强。坚持依法行政，主动接受人大法律监督、政协民主监督和社会舆论监督，办理市人大建议162件、市政协提案219件，办复率均为100%。政府职能进一步转变，完成“三张清单”编制，下放审批事项35项、取消23项，承接省里下放42项，调整10项。事业单位改革持续深化，精简机构42个。深入开展“三严三实”专题教育，严格落实中央八项规定，整改“四风”问题，干部作风进一步转变。全面推进“三公”经费电子监察，实施公务卡结算管理，“三公”经费下降26%。行政监察和审计监督成效明显，党风廉政建设和反腐败工作扎实推进。

　　2015年工作任务的完成，标志着“十二五”规划主要目标胜利实现。过去的五年是莆田发展极不平凡的五年，这是综合实力大幅提升，经济跨上新台阶的五年。全市生产总值比五年前翻一番，年均增长12.3%，增幅连续3年居全省第二位。财政收入、社会消费品零售总额、实际利用外资、外贸进出口总额分别是“十一五”末的2.4倍、1.9倍、1.6倍和1.4倍。规模以上工业企业数达1177家，规模工业产值翻一番。这是发展方式加快转变，产业转型升级取得显著成效的五年。农业发展质量和效益稳步提高。工业产业集群发展壮大，制鞋产业产值达700亿元，食品医药、工艺与创意产业产值均超过300亿元。第三产业占地区生产总值比重提升1.5个百分点，增幅连续3年稳居全省前3名，总量从全省第7位上升到第6位。万元GDP能耗下降16%。这是基础设施投资最多，城乡面貌翻天覆地变化的五年。全社会固定资产投资累计完成6064.3亿元，年均增长28.9%。城市建成区面积较五年前增加19.2平方公里。城乡一体化加快发展，基础设施全面改善，新增铁路里程78公里、高速公路里程193公里、城市道路里程251公里，公路网密度位居全省第一。港口总吞吐能力达3736万吨，是“十一五”末的2.34倍。新增电力装机容量173.1万千瓦，其中抽水蓄能120万千瓦、风电装机容量44万千瓦。荣获“国家园林城市”、“全国绿化模范城市”、“中国人居环境范例奖”、“省文明城市”和“省卫生城市”等称号。这是民生事业全面发展，人民生活水平显著提升的五年。新建公办幼儿园42所。新建扩建中小学校舍636幢，扩容城镇中小学32所，新增达标高中13所，中小学光纤接入全覆盖，城乡义务教育均衡发展。组建两家大型医疗集团，在全省率先实行新农合与城镇居民医保并轨运行，实现持卡就医实时结算，基本公共卫生服务均等化水平大幅提高。城镇登记失业率控制在3%以内，新增城镇就业10.1万人，转移农村劳动力19.6万人，城乡低保补助水平增长153%，6万人实现脱贫。城镇居民和农村居民人均可支配收入分别是“十一五”末的1.5倍和1.8倍。荣获“全国双拥模范城”四连冠。这是转变作风，为民务实提升服务的五年。全面推进依法治市，精简市级政府机构10个，整合12个。推行“三向并联”审批，市本级办结事项60多万件。扎实开展党的群众路线教育实践活动，整治“庸懒散奢”，效能问责2206人次。落实廉政建设责任制，惩治和预防腐败体系建设取得新进展。

　　各位代表，“十二五”取得的成就，是在省委、省政府和市委的正确领导下，全市上下解放思想、开拓进取的结果；与各级人大及人民代表的依法监督，各级政协、民主党派、工商联以及人民团体的民主监督和积极参与，驻莆部队、武警官兵的大力支持，港澳台同胞、海外侨胞的支持合作分不开的。在此，我代表市人民政府，向所有关心、参与和帮助莆田经济社会发展的同志们、朋友们，表示衷心的感谢！

　　我们清醒地认识到发展中存在的困难和问题：经济总量小，发展质量不高，创新能力弱；产业转型升级不快，高新产业、新兴产业比重低，生产性服务业发展滞后；部分企业经营困难，有的停产减产，防范金融风险压力加大；城镇化率还偏低，脱贫任务重，美丽乡村建设有待加快推进；环保压力大，节能减排面临新挑战；社会事业发展相对滞后，群众一些迫切需求尚未得到有效解决；人才引进、选拔、激励等政策和管理仍存在问题；政府职能转变还不到位，一些公务人员不作为、乱作为，少数人甚至违纪违法。对此，我们要坚持问题导向，以攻坚克难的精神加以解决。

　　二、认真实施“十三五”规划，全力建设宜业宜商宜居莆田

　　“十三五”时期是莆田加快发展、跨越发展的关键时期，是全面建成小康社会的决胜阶段。我们要树立强烈的忧患意识、机遇意识和追赶意识，凝心聚力，乘势而上，推动经济社会更好发展，让全市人民过上更加美好的生活。

　　“十三五”时期发展的总体要求：高举中国特色社会主义伟大旗帜，全面贯彻党的十八大和十八届三中、四中、五中全会精神，以马克思列宁主义、毛泽东思想、邓小平理论、“三个代表”重要思想、科学发展观为指导，深入贯彻习近平总书记系列重要讲话精神和对福建工作的重要指示，坚持全面建成小康社会、全面深化改革、全面依法治国、全面从严治党的战略布局，坚持发展是第一要务，着力创新发展、协调发展、绿色发展、开放发展、共享发展。认真落实中央支持海峡西岸经济区建设和福建加快发展的重大决策部署，按照建设机制活、产业优、百姓富、生态美的新福建要求，以保持经济稳定较快增长为目标，以转型升级为主线，以提高发展质量和效益为中心，把发展临港先进制造业作为支撑，实施“336”工程，培育产业龙头，壮大实体经济，加快形成引领经济发展新常态的体制机制和发展方式，全面推进经济建设、政治建设、文化建设、社会建设、生态文明建设和党的建设，努力建设宜业、宜商、宜居的莆田，力争提前全面建成小康社会。

　　“十三五”时期经济社会发展的主要目标：

　　——综合实力大幅提升。全市经济保持稳定较快发展，增幅高于全省平均水平1-2个百分点，地区生产总值年均增长10.5%左右，到2020年力争3000亿元，人均地区生产总值达到全省平均水平。全社会固定资产投资五年累计1万亿元以上，年均增长16%；财政总收入300亿元以上，年均增长10%左右，地方级收入力争200亿元，年均增长11%左右；服务业增加值比重力争40%，主要经济指标平衡协调，发展质量和效益明显提升。

　　——支柱产业基本形成。工业化和信息化融合发展水平进一步提高，制造业水平迈入全省先进行列，工业实力大幅提升。产业发展迈向中高端，集群化进程加快，龙头带动明显增强，化工（纺织化纤）新材料、食品加工、工艺美术、电子信息等产业成为福建先进制造业的重要基地，高端装备、医疗健康等新兴产业成长壮大。工业结构更加合理，工业发展整体竞争力提高。

　　——社会事业全面发展。就业、教育、文化、社保、医疗、养老、住房等公共服务体系更加健全，基本公共服务均等化水平稳步提高。社会就业更加充分，五年城镇新增就业人数9万人。社会保障实现全民覆盖。全面建成教育强市。基本医疗卫生服务保障体系更加完善。

　　——城乡区域发展更加协调。区域发展空间格局进一步优化，中心城区辐射带动作用进一步增强，城市建设管理水平进一步提升。新型城镇化试点示范带动作用增强，基本实现城乡发展一体化，到2020年户籍人口城镇化率48%左右，常住人口城镇化率67%左右。老少边岛地区发展步伐明显加快，实现“村村通快递”、“岛岛通班轮”。

　　——生态环境质量保持前列。生态文明示范区加快建设，单位地区生产总值能耗下降、二氧化碳和主要污染物排放总量减排完成省下达指标，土地、水、海洋等资源利用效率进一步提高。全面完成农村饮水安全工程。城市绿心有效修复合理利用，木兰溪、萩芦溪Ⅰ－Ⅲ类水质比例分别高于83.3%、95%。森林覆盖率稳定在60%左右，建成全国森林城市。

　　——人民生活水平全面提高。实现城乡居民收入增长和经济发展基本同步、劳动报酬增长和劳动生产率提高基本同步，居民人均可支配收入力争3.8万元，达到全省平均水平，提前比2010年翻一番。国家现行标准下农村贫困人口全部脱贫，空壳村全面消除。法治莆田建设全面推进，人民权益得到切实保障。社会治理能力和水平明显提升，市民文明素质和社会文明程度显著提高，包容和谐的社会基本形成，建成全国文明城市。

　　——改革创新取得重大进展。国家、省赋予的改革试点取得重大突破，新型城镇化、社会办医、电商示范城市等重点领域改革走在全国前列，可复制、能推广的试点经验更加成熟定型。市场活力、创新创业活力显著增强，基本形成适应创新驱动发展要求的制度环境，自主创新能力大幅提高，每万人口发明专利拥有量3件。全方位对外开放不断拓展，对台前沿平台和海丝节点城市作用更加凸显，对外开放的竞争优势进一步增强。

　　各位代表，以上奋斗目标，体现了省委、省政府对莆田发展的战略要求，表达了人民群众向往美好生活的共同愿望，展示了莆田加快发展的光明前景。我们坚信，经过五年不懈奋斗，一个综合经济实力显著增强、城市服务功能显著提升、社会文明程度显著提高、人民群众幸福感显著增加的宜业、宜商、宜居莆田，一定会崛起在海峡西岸！

　　三、稳中求进，积极作为，扎实做好2016年工作

　　今年是实施“十三五”规划的开局之年。做好今年工作，必须认真落实中央和省经济工作会议精神，认识新常态、适应新常态、引领新常态，坚持稳中求进的总基调，落实宏观政策要稳、产业政策要准、微观政策要活、改革政策要实、社会政策要托底的要求，去产能、去库存、去杠杆、降成本、补短板，增强持续增长动力。全市国民经济和社会发展的主要预期目标是：地区生产总值1850亿元、增长10.5%左右；规模以上工业增加值875亿元、增长10.5%；财政总收入200亿元、增长8%，地方财政收入125亿元、增长8%；全社会固定资产投资2080亿元、增长18%；社会消费品零售总额626亿元、增长12%；外贸出口总额33亿美元、增长5%；实际利用外资4亿美元、增长6%；居民人均可支配收入2.4万元、增长11%左右；落实节能减排任务。

　　实现上述目标，重点做好以下几个方面工作：

　　（一）扩大有效投资，促进经济稳定增长

　　进一步加大重点领域投资。实现年度固定资产投资2080亿元，其中产业类580亿元、基础设施类700亿元、社会事业类15亿元以上。港口领域，重点建设港航基础设施，连片开发莆头、东吴、罗屿作业区，续建国投湄洲湾煤炭码头装船泊位、湄洲湾航道三期，建成罗屿9-10号码头泊位。交通领域，重点开工建设福厦高铁客运专线、江涵大桥、纵三公路，推进“三纵一横三联”普通国省干线、滨海大道等建设，建成罗屿铁路支线，开展仙游至长乐城际轨道、涵江至江阴高速公路前期工作。能源领域，重点建设LNG 5-6号罐、湄洲湾第二发电厂、南日岛和平海湾海上风电，加快抽水蓄能电站二期前期工作。水利领域，重点除险加固东圳水库，建成乌溪水库，续建木兰溪防洪工程，开工建设宁海闸、西音水库、东圳水利枢纽引水配套工程。市政领域，重点建设木兰大道、白塘路、壶公路、八二一街跨木兰溪大桥，加快建设环湄洲湾尾水排海工程、妈祖城水厂、市第五水厂，抓好棚改项目，修建城市停车场、城市地下综合管廊。房地产领域，重点调整土地收储出让节奏，改造老旧小区，推广货币安置，打通商品房、安置房、保障房转换通道，化解库存，稳定市场。信息领域，重点完成城市公共信息平台、城市公共基础数据库、政务数据中心建设，新建互联网经济孵化器和创业园，加快建设智慧城市、电子商务国家试点城市。

　　深化项目招商引资。完善常态化招商机制，围绕三大支柱产业和三大新兴产业，分区域、分行业招商，坚持大中小配套、长中短衔接、政民外齐抓，突出高世代面板、CPL、食品加工产业链招商，推进电子信息、化工新材料等产业项目落地。深化民企项目招商，围绕产业链条缺失环节，加快在谈项目签约落地，促进医疗器械、汽车照明、再生纤维等新签约项目开工建设。

　　推动投融资体制改革和金融创新。深化投资审批制度改革，试行投资项目并联审查制度，减少前置审批，优化审批流程。改革基础设施领域投融资体制，加强政银企合作，建立政府和社会资本合作PPP项目库，提高融资性担保机构服务能力。积极争取国家专项资金，改造棚户区和市政基础设施，改善城乡供排水，推进新型城镇化。强化产业股权投资基金、产业互助成长资金的运作管理。创新信贷产品，用好增量信贷规模，盘活存量财政资金。依法处置信用违约，规范各类融资行为，坚决遏制非法集资，打击逃废债行为，防范化解金融风险，维护金融稳定。

　　（二）提高供给质量，厚植产业发展新优势

　　大力发展特色现代农业。落实最严格耕地保护制度，完成省下达粮食生产任务，发展设施果蔬、花卉、林业，新增设施蔬菜4000亩、文旦柚5000亩、油茶16000亩、林下经济8000亩，有效供给更加环保、营养、安全的农产品。继续建设“海洋牧场”，推动南日岛和后海现代渔业产业园发展，引导水产生态养殖，促进水产品加工流通，培育休闲渔业产业。推动农业规模经营，培育农业社会化服务组织，发展特色种养业、休闲农业、创意农业，增加农民经营性收入。认真做好第三次全国农业普查工作。

　　推进工业结构优化升级。着力加强供给侧结构性改革，优化存量，提升增量，全面落实《中国制造2025》行动计划，壮大支柱产业，培育新兴产业，提升传统产业，扩大有效供给，提高供给结构对需求变化的适应性和灵活性。实施产业龙头促进计划和降本增效专项行动，降低企业交易成本、税费负担、社会保障费、财务成本、电力价格、物流成本，提高企业市场竞争力。帮助有市场、有前景的企业渡过难关，对已停产半停产、连年亏损、资不抵债的企业，积极稳妥推进优胜劣汰、兼并重组，尽量减少破产清算。化工（纺织化纤）新材料产业重点推进CPL建成投产，打造合成纤维、合成树脂、清洁涂料产业链，加快建设巨型工程轮胎、子午线轮胎生产基地。食品加工产业重点推动啤酒、粮油食品精深加工等项目，带动食品加工产业链发展。工艺美术产业重点引导古典家具、珠宝首饰、油画、木雕等企业加大技术、品牌、管理和商业模式创新，高端产品树品牌、大众化产品拓市场，走差异化发展道路，增强市场竞争力。高端装备产业重点发展智能化数控机床、激光精密设备、新能源汽车等产业链，培育发展制鞋、纺织、木工等专用工业机器人，形成新增量。电子信息产业重点打造高世代面板、集成电路、光电通信等产业链，引导劳动密集型电子信息企业转型发展，形成产业链集聚效应。医疗健康产业重点推进医疗器械、生物制药、医疗耗材、医药物流等产业链建设，扩展普天药械网辐射范围，构建“一龙头三基地”产业格局。鞋服产业重点扶持企业开展“机器换工”，打通产品研发、生产、营销等关键环节，提升设计水平，发展高档、舒适和个性化产品，促进产业向价值链中高端发展。清洁能源产业重点发展LNG清洁能源，扩大海上风电规模，推进抽水蓄能电站建设。同时，扶持建筑业加快发展，提高市场占有率。

　　加快发展服务业。提升工艺城、珠宝城、博览城3个百亿市场，新增重点服务业企业50家，力争营业收入增长30%以上，新增限上商贸企业100家，力争商品销售额增长15%以上。建立临港大宗物品交易平台，推进普洛斯等10个重点物流项目，供给商业储备、仓储物流等服务产品。继续建设福建健康产业园等6个总部经济区。大力发展金融服务、研发设计等生产性服务业，培育新增长点。推进70个商贸重点项目，培育发展楼宇经济。发展农村电商，拓展“中国质造”系列活动，培育限上电商企业240家，实现销售额160亿元以上、零售额60亿元以上。推进全域旅游，创建湄洲岛、九鲤湖国家5A级和菜溪岩、瑞云山国家4A级景区，落实带薪休假制度，完善停车场、厕所、酒店等旅游设施，发展农家乐、渔家乐、森林人家，满足多层次旅游需求。

　　拓展发展新空间。完善莆田高新技术园区、木材贸易加工示范区、仙游、荔城和华林经济开发区等配套设施，实施“退城入园”，加快企业向园区集聚。高标准建设石门澳、兴化湾南岸、东吴、仙港、东峤、莆头等6个重点工业园区，重点完成道路、供水、污水处理等基础设施建设，提升园区承载力。续建兴化湾南岸一期填海工程，启动石门澳二期、海安海滨、前海围垦工程，促进产业集聚发展。实施“互联网+”行动计划，提升安福电商城、电商未来城、涵江电商产业园建设水平，发展药械、红木、银饰、房产、莆田名果等电商平台，打造跨境电商支付平台，支持仙游建设省级电子商务示范县、举办第二届中国红木精品博览会，促进互联网与鞋业、食品、工艺美术等特色产业融合发展。

　　（三）推进新型城镇化，促进城乡协调发展

　　强化中心城区建设。统筹规划、建设、管理三大环节，加强对城市的空间立体性、平面协调性、风貌整体性、文脉延续性等方面的规划和管控，留住城市特有的地域环境、文化特色、建筑风格等“基因”，全力以赴创建全国文明城市。加快发展中心城区和涵江、秀屿等区域中心，大力支持仙游发展，增强城市综合服务功能和辐射带动能力。保护修复和开发利用古谯楼、广化寺、萝苜田等历史建筑，建设历史文化街区和传统风貌区，改造城中村，保护文化遗迹，延续历史文脉，提升城市品位。治理木兰溪、城涵河道、北渠、下磨溪，启动南门、天九湾、莆田西片区等城市内涝治理，完成玉湖蓄涝区、东吴园区排涝工程。充分利用自然山体、河湖湿地、耕地、林地、草地等生态空间，提升城市地面蓄水、渗水和涵养水源能力，建设海绵城市。加快城市绿化美化花化彩化，保护荔林水乡特色，修建荔枝林带景观和凤凰山沿渠景观，构建绿道网络，打造绿色“慢生活”。促进城市治理现代化，建设数字城管，深化“两违”综合治理，加强滴撒漏执法，强化市容环境卫生整治。鼓励绿色出行，优先发展公共交通，修建公交站点，增建公共停车场，整治交通拥堵重点区域，让群众在城市生活得更方便、更舒心、更美好。

　　推进以人为核心的新型城镇化。深化户籍制度改革，完善居住证制度，加快本地人口市民化、外来人口本地化，把促进有能力在城镇稳定就业和生活的常住人口有序实现市民化作为重要任务。积极推进“多规合一”，全面开展城市设计。加快小城镇综合改革、“强镇扩权”改革和省级“小城市”培育等试点工作，提升榜头、黄石、西天尾、江口、埭头、枫亭等小城镇建设水平，加快建设南日岛，推广“幸福家园”试点，深化宅基地改革，促进农村产权交易流转。均衡配置城乡公共资源，推动公共服务向农村延伸。大力整治村容村貌，完善文化体育设施，建设美丽乡村。

　　推进精准扶贫。深入推进“五个一批”扶贫攻坚行动计划和“强村行动计划”，帮扶88个贫困村，消除集体经济空壳村50%以上、贫困人口2万人。继续实施造福工程，搬迁地灾点和偏远自然村。鼓励社会力量参与扶贫，发挥异地莆田商会助力扶贫作用。健全领导挂钩、部门帮扶、干部驻村、企业扶助制度，实施脱贫工作责任制，强化责任考核。

　　（四）深化改革开放，发展更高层次开放型经济

　　打造海丝节点城市。主动融入“一带一路”，密切海丝沿线经贸往来，推进高水平双向互动，做大双边贸易。组织油画、古典家具、珠宝银饰等文化产品赴沿线国家和地区展览展销。打造妈祖“海丝”文化品牌，构建多层次常态化交流合作机制。加强旅游合作，开发海丝沿线特色旅游产品。深化莆台合作，拓展对台小额贸易，吸引台湾企业来莆设立地区总部、配套基地、采购中心，举办第十一届海峡（莆田）工艺品博览会。建设世界妈祖文化中心，办好妈祖文化节、海峡论坛、妈祖春秋祭典等活动。争取列入赴台个人游试点城市。

　　深化重点领域改革。加快国家、省级改革试点工作。着力推进以满足新市民为出发点的住房改革，发展租赁市场，有效释放住房刚性需求和改善性需求。承接自贸区政策，推行第三方检验结果采信等10项改革事项。实行电子营业执照，加盖登记机关电子印章，赋予企业“电子身份”。率先开展电子商务统计试点工作。

　　大力推动创新创业。优化创新创业生态链，释放城市发展创新动能。培育创新主体，发展企业技术中心，实施重大成果转化工程、创新驱动助力工程和“金桥工程”，形成一批创新型企业群、高新技术企业群。统筹建设各类人才队伍，培养引进创新型、高技能和高层次人才，完善和落实人才政策，营造人才辈出、人尽其才的社会环境。发展科技服务新业态，扶持科技企业孵化器建设，集聚创新资源，发展创业投资，培育为创新服务的股权众筹、互联网金融等新型业态；发展众创、众包、众扶等新型服务空间，建设1个县级创业创新示范基地、2个市级众创空间。

　　促进外经贸转型升级。完善外经贸扶持体系，培育外贸新型业态，实行“一对一”重点出口企业重点扶持制度，组织企业参加广交会、商交会、华交会等展销会，支持企业参与国家援外项目。完善引进外资项目服务机制，保护知识产权和企业合法权益。创新口岸通关模式，建设“单一窗口”通关一体化信息平台。

　　（五）坚持绿色发展，促进人与自然和谐共生

　　深化环境治理。深入实施大气、水、土壤污染防治行动计划，控制主要污染物排放。实施环保网格化监管，健全环境风险防范与应急处置体系。抓好火电厂脱硫脱硝，改造小锅炉，淘汰黄标车，整治机砖瓦窑、石材加工、汽车维修等环境问题突出行业。实施中心城区雨污分离，强化污水管网、污水处理厂管理，提高污水收集率。完善生活垃圾分类处理机制，建成生活垃圾焚烧发电厂三期，推进仙游循环再生能源发电厂、餐厨垃圾处置场、危险废物处置中心等环保基础设施建设。

　　筑牢生态安全屏障。实行最严格的环保制度，建立生态环境红线，完善环境风险源监控、风险评估、生态补偿机制，继续实施三沿一线、一重山天然林资源保护等重大生态修复工程。推进绿心的保护性开发。修建土海、白塘湖、延寿溪湿地、湄洲岛红树林公园，保护木兰溪源、老鹰尖省级自然生态区。实施“四绿”工程，完善沿海防护林体系，建设滨海生态走廊，创建国家森林城市。建设安全生态水系，维持木兰溪、萩芦溪下游生态需水量，深化东圳水库水环境综合治理，建设金钟、外度水库等饮用水源保护区，让市民喝上放心水。

　　推动低碳循环发展。改造LNG循环产业园，新建莆田市循环经济园、建筑产业现代园，实现产业废物交换利用、能量梯级利用。强化主要污染物减排等约束性指标管理，建立倒逼传导机制。培育非化石能源产业，抓好重点行业节能减排工作，淘汰落后产能。

　　（六）共建共享发展，让全市人民有更多获得感

　　优化公共服务。优先发展教育，扩大普惠性幼儿园覆盖面，强化幼儿园规范化建设。推动义务教育均衡发展，加强教师队伍建设，促进城乡教师交流，扩容城区义务教育学校，新增学位8000个。支持和规范民办教育，发展现代职业教育，培养“336工程”急需人才。加快莆田学院、湄洲湾职业技术学院迁建。建成“三馆一宫”，迁建市档案馆。举办第一届市艺术节。支持文艺创作，实施莆仙戏振兴计划。完善城乡公共体育设施，广泛开展全民健身活动，办好第25届世界技巧锦标赛。加强计划生育管理和服务，落实一对夫妇生育两个孩子政策，严厉查处政策外多孩生育，促进人口均衡发展。繁荣发展社会科学、新闻出版、广播影视、文学艺术、地方志等事业。

　　提高医疗卫生水平。优化基层医疗服务，改善提升社区、乡镇医院条件，构建布局合理、分工协作的医疗卫生服务体系和分级诊疗体系。实行“三医”联动，改革药品集中招标体制，完善公立医院收入分配、绩效考核、医生多点执业机制。加强医疗人才队伍建设，加大妇产和儿科投入，提高全科医生、家庭医生服务能力。完成市第一医院扩建、仙游妇幼保健院迁建，续建市中医医院，开工建设市肿瘤医院、仙游医院新院区、涵江国药医院、北岸医院、新安国际医院和艾普强质子医院。创建国家卫生城市。做好社会资本办医试点，举办医疗健康产业投资洽谈会。全面防控传染病、地方病，预防消除职业病。实施食品安全战略，构建社会共治体系，让人民群众吃得放心。

　　加强社会保障。强化社会政策托底功能，健全就业服务体系，新增城镇就业2万人，转移农村劳动力3万人，城镇登记失业率控制在3%以内。开展全民参保登记计划试点和机关事业养老保险制度改革，将外来人口和失地农民纳入城镇职工养老保险体系。积极推进国家级工伤预防试点，将公务员、参公单位人员纳入工伤保险。实施城乡居民大病保险制度，改革医保支付方式，实行医保同城化。健全工资水平正常增长机制、支付保障机制，完善最低工资保障机制，增加城乡居民收入。统筹救助体系，发展慈善、福利和残疾人事业，保障困难群众基本生活。鼓励社会力量参与养老事业，提高专业化养老服务水平。

　　创新社会治理。完善立体化社会治安防控体系，建立市级人民调解指挥平台，落实重大决策社会稳定风险评估制度，健全社会矛盾排查预警和调处化解机制。实施“七五”普法，加强法律援助。落实责任信访，推进法治信访，维护群众合法权益。严密防范、惩治违法犯罪活动，维护市场经济秩序和群众合法权益。完善公共信用信息、信用评价、信用惩戒机制，创建“信用莆田”。规范管理社会组织，加强城乡社区建设。健全公共安全体系，实行党政同责、一岗双责、失职追责，落实企业主体责任，遏制各类事故发生。加强防汛防台风工作，完善防灾减灾救灾体系，提高自然灾害防御能力和突发事件应对能力。加强海防、人防工作和民兵、预备役建设，做好“双拥”工作。支持工青妇等人民团体工作，强化外侨、民族宗教等工作。

　　办好为民实事。加大民生投入，今年全市安排民生支出122亿元，着力办好东圳水库水环境综合治理工程等18项为民实事。

四、全面建设法治政府

　　坚持党的领导，把政府工作纳入法治轨道，通过法律和政策向社会传导正确价值取向，推进全民守法。

　　（一）把职能科学、权责法定落到实处。坚持在市人大及其常委会的法律监督和政协的民主监督下依法开展工作。进一步转变政府职能，提高行政效能和服务水平。做好政府换届工作。健全依法决策机制，依法制定政府规章。深化审批改革，落实“三张清单”，完善网上办事大厅，推广“互联网+政务服务”，使政务服务更为高效透明、公平可及。建立全市统一的公共资源交易平台，清理规范涉企收费，推进产权保护法治化。健全领导干部学法制度，促进行政复议规范化。

　　（二）把执法严明、公开公正落到实处。改进市、县区（管委会）政府工作体制机制，做到分工明确、责任清晰，运转高效。改革综合行政执法体制，完善执法争议协调机制，严格规范公正文明执法，切实维护群众合法权益。加大食品药品、安全生产、质量监督、城市管理、海洋渔业、知识产权等重点领域执法力度，依法惩处违法行为。加强行政执法监督，规范执法行为，提升政府法治形象。

　　（三）把廉洁高效、守法诚信落到实处。深入落实党风廉政责任制，主动接受司法监督、社会监督和舆论监督，强化重点领域和关键环节监察、审计，坚决查处违法违纪行为。按照“三严三实”要求，持之以恒纠正“四风”，坚持“四下基层”、“马上就办”。项目招商、落地建设、解决问题做到一天都不耽搁。提高政策执行力和工作落实力，保持敢于担当、奋发有为的精神状态。严肃财经纪律，厉行勤俭节约，完成公务用车制度改革，确保“三公”经费只减不增。建设诚信政府，促进社会诚信提高。

　　各位代表，宏伟目标的实现需要共同努力和合力攻坚。让我们更加紧密团结在以习近平同志为总书记的党中央周围，在中共莆田市委的领导下，团结依靠全市人民，融入新常态，把握新机遇，为加快建设宜业、宜商、宜居的莆田而努力奋斗！
